# Electrochemical Sensing of Lead in Drinking Water Using Copper Foil Bonded with Polymer

**DOI:** 10.3390/s23031424

**Published:** 2023-01-27

**Authors:** Taufique Z. Redhwan, Younus Ali, Matiar M. R. Howlader, Yaser M. Haddara

**Affiliations:** Department of Electrical and Computer Engineering, McMaster University, 1280 Main Street West, Hamilton, ON L8S 4K1, Canada

**Keywords:** electrochemical sensor, lead detection, rolled-annealed copper, polymer, surface activated bonding, water quality monitoring

## Abstract

Levels of lead (Pb) in tap water that are well below established guidelines are now considered harmful, so the detection of sub-parts-per-billion (ppb) Pb levels is crucial. In this work, we developed a two-step, facile, and inexpensive fabrication approach that involves direct bonding of copper (Cu) and liquid crystal polymer (LCP) followed by polyester resin printing for masking onto Cu/LCP to fabricate Cu thin-film-based Pb sensors. The oxygen plasma-treated surfaces resulted in strongly bonded Cu/LCP with a high peel strength of 500 N/m due to the highly hydrophilic nature of both surfaces. The bonded specimen can withstand wet etching of the electrode and can address delamination of the electrode for prolonged use in application environments. The Cu-foil-based electrochemical sensor showed sensitivity of ~11 nA/ppb/cm^2^ and a limit of detection (LOD) of 0.2 ppb (0.2 µg/L) Pb ions in water. The sensor required only 30 s and a 100 µL sample to detect Pb. To date, this is the most rapid detection of Pb performed using an all-Cu-based sensor. The selectivity test of Cu to Pb with interferences from cadmium and zinc showed that their peaks were separated by a few hundred millivolts. This approach has strong potential towards realizing low-cost, highly reliable integrated water quality monitoring systems.

## 1. Introduction

Lead (Pb) is one of the toxic heavy metals that accumulates in water, causing adverse effects on human health [1,2]. Pb concentrations previously considered safe are now believed to cause neurodevelopmental and behavioral disorders in children [3,4,5] and increased blood pressure and renal dysfunction in adults [6,7]. Long-term exposure to Pb may cause cancer in the kidneys, lung, or brain [7]. Thus, careful monitoring of its levels in tap water is urgently needed. The Canadian Federal-Provincial-Territorial Committee on Drinking Water (CDW) has recently proposed updating the existing World Health Organization (WHO) guideline on the safe limit for Pb in drinking water from 10 µg/L (10 ppb) to 5 µg/L (5 ppb) [7,8]. Although directives of different regulatory bodies have different thresholds for Pb levels, they were established based on analytical limitations and feasibility. Levels under which Pb can no longer cause adverse health effects are now difficult to identify [7] since these effects do not show up immediately and depend on factors such as exposure time and human age. Thus, it is desirable to detect Pb at the lowest concentration possible. The proposed CDW guideline also emphasizes the easy identification of contaminant levels across distributed water sources. The conventional lab-based Pb detection techniques such as atomic absorption spectroscopy [9] and inductively coupled plasma mass spectrometry [10] are capable of detecting trace amounts of Pb precisely; however, they are bulky, complex, expensive, and time-intensive. In contrast, electrochemical sensors are ideal for on-site water quality monitoring as they are low-cost, easy to fabricate, handy, and offer ppb limits of detection (LOD) [11,12].

In recent years, considerable work has been performed on electrochemical sensors for Pb detection using a wide range of electrode materials, including graphene oxide (GO) [1,13,14,15], mercury (Hg) [16], bismuth (Bi) [17,18,19], bimetallic mercury–bismuth with carbon nanotubes [20], β-cyclodextrin-modified multiwall carbon nanotubes (MWCNTs) [21], boron-doped diamond with nanoparticles (NPs) [22], silver (Ag) [23], gold (Au) with ruthenium–GO composite [24], gold-black phosphorus (BP) nanosheet field effect transistors (FETs) [25], platinum (Pt) [26], metal oxides [27], bismuth with silica NPs [28], carbon [29] and copper [30]. The sensors based on graphene oxide [1,13,14,15], MWCNTs [21], and nanocomposites [20,24] are miniaturized and can detect ppb levels of Pb due to their high effective surface area and catalytic properties, and possess good stability. However, there are several challenges of using these nanomaterials including nanomaterial synthesis cost, toxicity, and disposal. Moreover, the modification of the bare electrode, for example, glassy carbon electrode (GCE) [1,13,14,15,20,28], screen-printed carbon electrode (SPCE) [21] or Au electrode [24] are fabricated by drop casting with functional nanomaterials, which are attached to the electrode by a weak Van der Waals force. Therefore, they are inappropriate for online tap water and river water monitoring due to the delamination of functional materials from the bare electrode. Furthermore, the drop casting process is less controllable and does not offer batch fabrication, resulting in significant variations in film thickness and exposed electrode area, making it unsuitable for mass fabrication.

Improved sensitivity of a bare mercury (Hg) electrode towards lead was reported by using copper in [16]. However, the sensors can detect ppb of Pb, and mercury itself is very toxic. Additionally, a fresh copper–mercury film is required to achieve good reproducibility, making it unsuitable for remote water monitoring. The bismuth-based electrode reported in [17,18,19] detected ppb of Pb and offered good substrate adhesion with the electrode but separate working, counter and reference electrodes are required for sensing, making it inappropriate for developing integrated sensing systems. Black phosphorus (BP) nanosheet field effect transistors (FET) with gold electrodes offered good sensitivity and integration capabilities [25]. However, the sensor’s fabrication process is complex and expensive. Moreover, the adhesion between the BP nanosheet electrode is not strong, and the sensor’s stability is short due to the use of BP nanosheets. Techniques such as drop casting, e-beam evaporation, vacuum sputtering, or in situ plating can sometimes limit the thickness of electrodes to thin films (<1 µm) that often degrade or delaminate from the substrate due to poor adhesion or sensing in acidic electrolyte [2,19,26,31]. The substrate adhesion can be improved by depositing a titanium (Ti) underlayer [31], but this also increases the complexity and cost of the process, and may compromise the sensor’s longevity due to delamination of the adhesion layer for prolonged use. Simpler fabrication processes, such as screen printing, can produce thick-film electrodes (>1 µm), but in most cases, the inks used in screen-printed electrodes contain some non-conductive binders or insulating polymers to improve substrate adhesion [32]. These binders increase the electron transfer resistance between electrodes and slow down the reaction kinetics, thus also affecting sensor performance.

An integrated copper (Cu)-foil-based electrochemical sensor can address the challenges associated with detecting low levels of lead (Pb) in water. Copper has high conductivity and a good affinity for Pb, making it a suitable choice for sensors [30]. Additionally, it is cheaper and more compatible with different fabrication approaches than other materials such as gold or platinum. In this work, we use rolled-annealed (RA) Cu to produce >10 µm thick foil electrodes, which have a compact grain structure [33] and larger cross-sectional area that improves conductivity and signal integrity [34,35]. This facilitates the flow and detection of low electrolytic cell current during trace analyte detection, which can reduce the sensor’s response time [17]. However, these benefits of RA Cu electrodes can be offset by their difficulty in bonding with other materials due to poor adhesion properties and lack of mechanical interlocks [36]. Strong adhesion and bonding between Cu and polymer are necessary to prevent electrode delamination during fabrication and sensing [31,37].

In this paper, we fabricated Cu-foil-based electrochemical Pb sensor without nanomaterials and conventional lithography. We used surface activated bonding to attach the RA Cu foil with LCP [2,38] at room temperature, followed by heating at 230 °C under 0.3 MPa, resulting in a strongly bonded Cu/LCP interface. Then, this specimen was directly fed into an inexpensive LaserJet printer to print a polyester resin-based electrode mask on the bonded Cu, followed by etching that resulted in a Cu-based robust Pb sensor on LCP. This approach is facile, low-cost, and swift, and it offers mass production of Pb sensors. We calibrated the sensors using an anodic stripping voltammogram and evaluated their sensing performance. These Cu-foil-based sensors could be the basis for rapid detection of sub-parts-per-billion level Pb in water.

## 2. Materials and Methods

### 2.1. Reagents

Electrochemical sensing of Pb is based on a redox process that requires a supporting electrolyte. In our experiments, we used sodium acetate buffer (0.2 M, pH 5.2) solution. The buffer was deoxygenated for 120 s through nitrogen bubbling to eliminate the effects of dissolved oxygen on Pb sensing peaks. A stock solution containing 10 mM Pb was prepared by dissolving 828 mg of lead (II) nitrate (Prod. ID 228621, MilliporeSigma, Burlington, MA, USA) in 250 mL of DI water. The stock solution (or nearest lower concentration) was then diluted with deoxygenated acetate buffer in a 1:9 *v*/*v* ratio. For example, 1 mL of 1 mM Pb solution was mixed with 9 mL of acetate buffer to obtain 100 µM Pb. Solutions containing 10 µM, 1 µM, 100 nM, 10 nM, and 1 nM Pb concentrations were prepared for sensor calibration. Additionally, the interference effects of Cd and Zn were investigated because they commonly co-exist with Pb in surface water [30]. For this, cadmium nitrate (Prod. ID 642045, MilliporeSigma) and zinc nitrate (Prod. ID 228737, MilliporeSigma) were used. Cu etchant was prepared from 1.0 M HCl and 30% H_2_O_2_ in a 1:1 *v*/*v* ratio. The role of hydrogen peroxide in this mixture is to speed up Cu etching. We used 1.0 M KCl electrolyte to chlorinate Cu foil for fabricating the Cu/CuCl_2_ reference electrode. A polydimethylsiloxane (PDMS)-based sensor encapsulant was prepared using Sylgard 184 (Dow Corning, Midland, MI, USA) silicone elastomer kit by mixing the base/curing agent in a 10:1 *v*/*v* ratio.

### 2.2. Sensor Fabrication

For the fabrication of the sensor, we used a two-step, facile, and inexpensive approach that involved the direct bonding of 50 µm-thick rolled-annealed (RA) Cu foil and 50 µm-thick liquid crystal polymer (LCP) substrate (Vecstar CTX-100, Kuraray, Tokyo, Japan) using surface-activated bonding (SAB) [38]. The bonding was achieved by pre-cleaning the Cu and LCP specimens with acetone, then exposing each to radio frequency (RF) oxygen reactive ion etching (O_2_-RIE) plasma for 240 s. The activated surfaces were then contacted, followed by heating at 230° C under 0.3 MPa to bond the Cu/LCP. Table 1 lists the optimized SAB process parameters used in this work to achieve high bonding strength. This SAB process is a further development of our previous work where we used either two-stage plasma activation [2] or high RF power (~720 W) and high contact pressure (~806 MPa) [39,40,41]. Finally, the Cu side of the bonded specimen was polished using 800/2400 grit SiC paper to remove the oxide layer on the Cu side that formed due to thermal oxidation in air.

**Figure 1:**
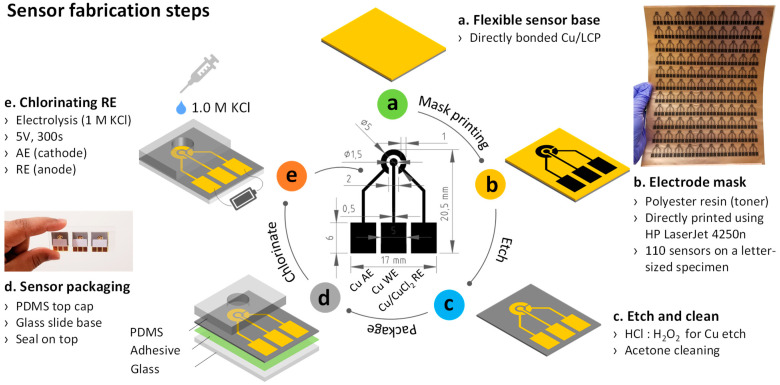
(**a**–**e**) Pb sensor fabrication steps. Inset illustrates the electrode size. A letter-sized flexible specimen can accommodate 10 × 11 sensors. SAB parameters used for direct bonding of Cu/LCP specimen are listed in Table 1.

Figure 1a shows the surface-activated bonding of Cu/LCP that was used to fabricate three electrodes: the auxiliary electrode (AE), working electrode (WE), and reference electrode (RE). The WE and the CE sustain the cell current, while the RE provides a reference for the measurement and control of the potential of the WE. This is necessary to detect the electrochemical signature of Pb, which is a current peak registered at a specific potential. Three electrodes were achieved by directly printing the all-electrode pattern on the Cu side of the Cu/LCP bonded specimen using an HP LaserJet 4250n printer (Figure 1b). The polyester resin-based printer toner served as the protective mask during wet etching of the Cu electrodes. After etching the Cu, the mask was wiped off using acetone, followed by rinsing in DI water for 30 s, resulting in a sensor with integrated three electrodes (Figure 1c). The polymer side of the sensor base was then attached to a clean glass slide using epoxy adhesive (Figure 1d). A standard single-hole puncher was used to create a 6-mm-diameter hole through the center of a 17 × 15 mm^2^-sized, 4-mm-thick PDMS encapsulant (separately prepared). The encapsulant hole was then aligned with the electrodes and bonded to the base specimen using plasma discharge and transparent epoxy [42] (Figure 1d). Next, the RE was chlorinated by adding 1.0 M KCl into the PDMS well and setting up an electrolytic cell between the RE (anode) and AE (cathode) (Figure 1e). The conversion of Cu RE to CuCl_2_ was performed by the application of a fixed +5.0 V potential to this cell for 300 s. Finally, the sensor was briefly rinsed in DI water, dried, and stored in a sealed container before use.

### 2.3. Electrode Characterization

We investigated the bond strength of directly bonded Cu/LCP (cut into 10 mm wide strips) using a Shimadzu AG-X tensile peel tester. The peel rate was 30 mm/min. This 180-degree peel test measured the substrate adhesion that is integral to the reliable performance of the electrodes [31]. The bond mechanism between Cu and LCP was investigated by studying the changes in surface properties (hydrophilicity and roughness). The water contact angle measurements were conducted using the sessile drop method (drop shape analysis systems DSA100, KRÜSS, Hamburg, Germany) with a 6 µL de-ionized water droplet. The surface roughness of 1 × 1 µm^2^ Cu and LCP were measured using an atomic force microscope (AFM, Dimension Icon, Bruker, Billerica, MA, USA) with an etched silicon tip tapping at 0.977 Hz. Finally, the surface quality of Cu/LCP bonding side was observed using a scanning electron microscope (JSM-7001F, JEOL, Tokyo, Japan).

### 2.4. Voltammetry Experiments

A miniature USB-powered potentiostat (EmStat3, PalmSens, Houten, The Netherlands) with PSTrace data analyzer software was used in electrochemical experiments. In each experiment, measurements were made on a fresh sensor probed to the EmStat3 so that residual effects could be avoided. A 100 µL sample was placed in the PDMS well. This electrolytic chamber was then covered with a 22 × 22 mm^2^ glass slide to minimize sample oxidation during voltammetry. Cyclic voltammetry (CV) was initially repeated three times in acetate buffer (0.2 M, pH 5.2) with 1 mM Pb to determine the oxidation and reduction peak positions of Pb. In cyclic voltammetry, the potential of the WE is ramped linearly versus time. After the set potential is reached, the WE potential is ramped back to the initial potential in the opposite direction. This way, it is possible to control the redox behavior of a species that is adsorbed onto the electrode. The CV scan rate was 100 mV/s, and the CV potential sweep range was −0.8 V to 0 V. Square wave anodic stripping voltammetry (SW-ASV) is popular for fast detection of analyte and was used to record current peaks corresponding to different concentrations of Pb in buffer. These data were used to calibrate the sensor. For stripping voltammetry, a −0.6 V deposition potential was first applied on the Cu WE for 10 s to reduce Pb^2+^ ions to Pb^0^. The electrodeposited Pb atoms were then stripped from the surface of the Cu WE by sweeping the electrode potential from −0.8 V to −0.2 V. The SW-ASV settings were 10 mV, 100 mV, and 10 Hz for step potential (increment), pulse amplitude (half peak-to-peak value), and frequency, respectively. Optimization of these settings is discussed in the next section.

## 3. Results and Discussion

### 3.1. Directly Bonded Cu/LCP for Sensing Electrodes

**Figure 2:**
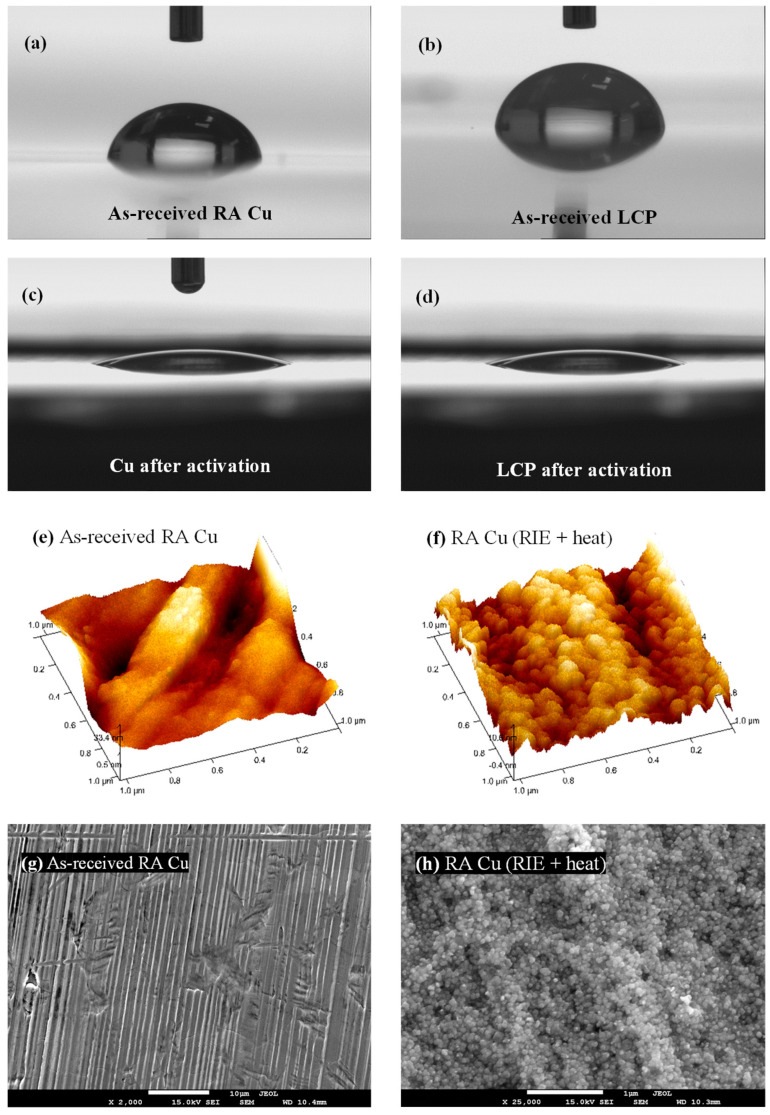
(**a**–**d**) Water contact angles (using 6 µL water drop, viewing angle = 4°) of: (**a**) as-received RA Cu (~79°), (**b**) as-received LCP (~83°), (**c**) activated RA Cu (~13°), and (**d**) activated LCP (~11°). The O_2_-RIE plasma treatment (Table 1) made Cu and LCP highly hydrophilic. (**e**,**f**) Surface morphology of 1 × 1 µm^2^ as-received RA Cu before and after plasma (followed by heat) treatment. (**g**,**h**) SEM image of RA Cu before and after plasma treatment.

The SAB process resulted in a strongly bonded Cu/LCP interface that did not delaminate during sensor fabrication or sensing. The peel strength of the interface was 500 N/m. The bond formation was investigated in terms of the changes in surface hydrophilicity and roughness. The hydrophilicity of a surface is determined from its water contact angle [43]. Highly hydrophilic surfaces (<<45°) are usually easier to bond [44]. As Figure 2a,b show, the as-received Cu and LCP are initially hydrophobic (>45°) with contact angles of 79° and 83°, respectively. This is due to the benzene and naphthalene groups of the LCP and the native oxide on Cu, all of which have low chemical affinity to hydroxyl (OH^−^) groups in water [45]. The O_2_−RIE plasma activation results in highly hydrophilic Cu and LCP surfaces as confirmed by their low contact angles of 13° and 11°, respectively (Figure 2c,d). This indicates the formation of OH^−^ groups at the dangling sites of oxide and carbonyl groups of Cu and LCP [2,39,45].

These OH^−^ chains condense through dehydration when the contacted Cu and LCP are heated under external pressure. This forms an intermediate oxide layer [2] that primarily develops on the Cu side due to thermal oxidation in air. To observe the effect of heat on the activated Cu foil alone, Cu was heated without any LCP contact, and the root mean square (rms) surface roughness was measured. Heating a plasma-treated Cu surface produces Cu_x_O nanoparticles that contribute to a reduction in the surface roughness of Cu foil from 8.91 nm (Figure 2e) to 3.08 nm (Figure 2f). This is desirable since smooth surfaces offer effective bonding in the SAB process [38]. The thermally grown nanoparticles on the plasma- and heat-treated Cu surface can be seen in the scanning electron micrograph (Figure 2h). These nanoparticles may also fill up the micro-gaps of the electrode-substrate interface and eventually diffuse into LCP [39] under the bonding conditions (Table 1). Therefore, the Cu/LCP bond may be formed by a combination of hydrophilic bonding [46] and nanoparticles interdiffusion [39].

### 3.2. Cu-Based Sensing Electrodes

**Figure 3:**
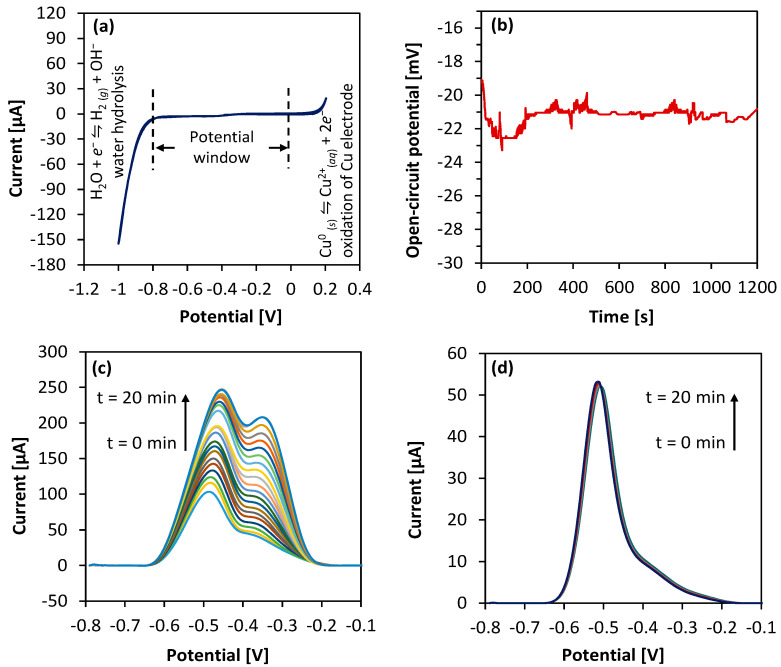
(**a**) Cyclic voltammogram of fabricated Cu WE in blank (no added Pb) acetate buffer. Scan rate was 100 mV/s, sweep voltage = −1 V to + 0.2 V. (**b**) Open-circuit potential response of fabricated Cu/CuCl_2_ RE measured against a commercial Ag/AgCl RE in acetate buffer. (**c**) ASV of 1× non-sealed Cu-based sensor with 1 mM Pb in non-deoxygenated buffer taken over 20 min repeatedly. (**d**) ASV of 1× sealed Cu-based sensor with 1 mM Pb in deoxygenated buffer taken over 20 min repeatedly. For ASV parameters, refer to Table 2.

Here, we investigated the potential window of the Cu WE by looking at its electron transfer during Pb oxidation that can result in a current peak. The idea here is to make use of the flat current response of the WE in a blank solution (no Pb) at these potentials. We identified this potential window by performing cyclic voltammetry of the Cu WE in blank acetate buffer electrolyte. The CV scan rate was 100 mV/s, and the potential sweep range was −1 V to +0.2 V. Voltammetry was performed against a standard Ag/AgCl RE and Pt AE. As shown in Figure 3a, the Cu WE offers a potential window from −0.8 V to 0 V where the characteristic Pb oxidation peak is produced, hence the use of the Cu WE is justified. At potentials more negative than −0.8 V, water hydrolysis produces bubbles of hydrogen gas on the Cu WE that disrupts the flow of electrons between the AE and the WE, thereby increasing the resistance in solution and causing a loss of WE potential control. This eventually degrades the WE. In addition, at potentials more positive than 0 V, the Cu WE oxidizes and loses sensitivity due to heavy oxidation.

For the auxiliary electrode, an inert material such as Pt is usually preferred. This is because a current flow between the AE and WE tends to force a reduction reaction which is offset by a change in AE surface potential. However, this change in surface potential is undesirable as it results in unstable electrochemical cell conditions. For this reason, AEs are made of highly polarizable material such as Pt to prevent potential drifts. Cu, on the other hand, is non-polarizable, and it has been demonstrated that Cu AE can maintain a stable potential long enough (up to an hour for electrodes thicker than 1 µm) [30]. In this work, we used thick-foil (>10 µm) Cu electrodes for fabricating a disposable sensor, so the cell stability should not be a major concern.

We next investigated the feasibility of the fabricated Cu/CuCl2 RE by measuring its open-circuit potential with respect to a commercial Ag/AgCl RE over 20 min. As Figure 3b shows, the potential response of Cu/CuCl_2_ remains stable over 1200 s with a potential drift of <5 mV. This is adequate for a quasi-reference electrode and is acceptable when compared to its miniaturized form factor, fabrication complexity, and cost. The variation in open-circuit potential could be due to slow dissolution of CuCl_2_ in buffer, as is also the case with commercial Ag/AgCl electrodes [47]. The most notable challenge faced during RE miniaturization is the rapid dissolution of small electrode volumes (such as Ag and AgCl) which leads to shorter lifetimes [47]. A thin layer of reference electrode undergoes depletion faster and may produce mixed potential response due to any metallic adhesive underlayer or terminal that becomes exposed. We have overcome both limitations (rapid dissolution and interference) by replacing thin-film electrodes with all-Cu-based thick-foil electrodes.

We further investigated the effect of Cu/CuCl_2_ dissolution in acetate buffer. In Figure 3c, ASV was repeated on a single sensor (non-sealed) for 20 min using the same test solution containing 1 mM Pb in non-deoxygenated buffer. The voltammogram shows a gradual increase in the shoulder peak at −330 mV and a shift of Pb oxidation potential from −500 mV to −440 mV. In contrast, the shoulder peak and potential shift were not observed when the same experiment was repeated with a sealed sensor and 1 mM Pb in deoxygenated buffer (Figure 3d). These observations indicate that for an experiment lasting at least 20 min, the sensor repeatability for a single experiment is affected by dissolution of Cu/CuCl_2_ from the RE under the corrosive influence of dissolved oxygen in buffer and from the ambient environment. However, these issues can be addressed by using deoxygenated buffer and glass-sealing as demonstrated in this work.

### 3.3. Optimization of Sensing Parameters

**Figure 4:**
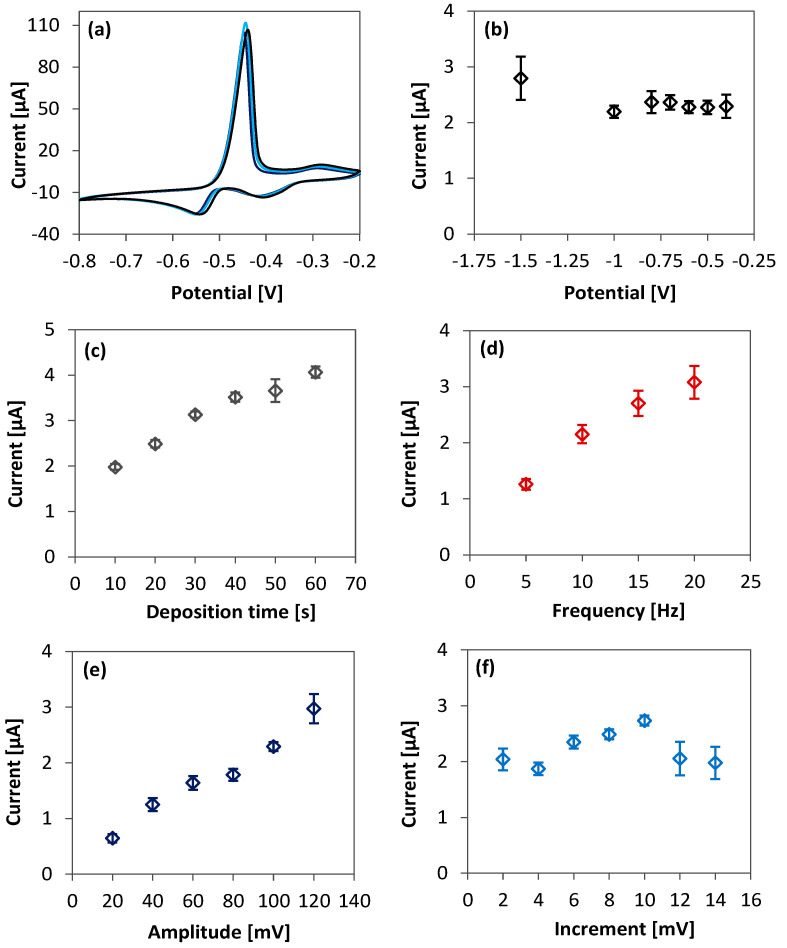
(**a**) CV in 0.2 M, pH 5.2 acetate buffer with 1 mM Pb. Scan rate = 100 mV/s; sweep voltage = −0.8 V to −0.2 V. (**b**–**f**) Optimization of ASV parameters with 10 µM Pb in buffer. Optimized values were −0.6 V, 10 s, 10 Hz, 100 mV, and 10 mV for deposition potential, deposition time, square-wave frequency, amplitude, and increment step-size, respectively (except for the parameter being varied, other related ASV parameters follow Table 2 during each optimization). Error bars obtained from 10 repeats (n = 10).

Figure 4a shows the cyclic voltammetry (CV) characteristic behavior of 1 mM Pb in acetate buffer (0.2 M, pH 5.2). The CV results show two sets of peaks for both Pb deposition (reduction of Pb^2+^ to Pb^0^) and Pb oxidation (from Pb^0^ to Pb^2+^). In both cases, the double peaks occur because of a high concentration of Pb [30]. The smaller peaks are due to a thin layer of Pb atoms directly deposited on the surface of the Cu WE. The larger peaks are due to further deposition of Pb on the already present Pb layer instead of the Cu electrode. Therefore, the peak at −413 mV (−8.7 µA) corresponds to Pb deposition on the Cu electrode, followed by thickening of the deposition layer at −552 mV (−17.7 µA). During oxidation, the thick layer of Pb first depletes at −447 mV (103.7 µA) and finally at −270 mV (8.9 µA) where Pb atoms are removed from the electrode. After identifying the peak positions from CV, we optimized the SWASV parameters (deposition potential, time, and stripping waveform settings) to obtain a good trade-off between low signal variation, rapid detection, and maximum peak current.

Deposition potential is one of the important parameters for ASV, and its value is often selected at 300−500 mV more negative than the standard reduction potential *E*^0^ of the metal to be reduced (*E*^0^ for Pb^2+^ + 2*e^−^* ⇋ Pb_(*s*)_ is −0.126 V) [48]. To understand this optimization, we begin by considering the Nernst equation [48] that relates the concentration of the Pb^2+^/Pb^0^ redox couple on the working electrode (surface concentration, [*Pb*^2+^]*_electrode_*) and the applied potential, E (Equation (1)).

(1) $$E=E_{Pb^{2+}/Pb\;}^{0}-\frac{0.05916}{2}log\frac{1}{{\left[Pb^{2+}\right]}_{electrode}}$$

If we choose a quantitative reduction of 99.99% *Pb*^2+^ to *Pb*^0^, then the surface concentration of *Pb*^2+^ is related to the known bulk concentration of *Pb*^2+^ in buffer, ${\left[Pb^{2+}\right]}_{bulk}$, by:

(2) $${\left[Pb^{2+}\right]}_{electrode}=0.0001\times {\left[Pb^{2+}\right]}_{bulk}$$

Rewriting Equations (1) and (2), the minimum potential required for quantitatively reducing 1 nM *Pb*^2+^ to *Pb*^0^ can be calculated (Equation (3)).

(3) $$E=-0.126-\frac{0.05916}{2}log\frac{1}{0.0001\times \left(1\times 10^{-9}\right)}=-0.51\;V$$

To experimentally optimize the deposition potential used, we tested different values from −0.4 V to −1.5 V and recorded the ASV peak currents (Figure 4b). From −0.4 V to −0.6 V, the peak current was almost unchanged, however the variation (indicated by error bars on the graph) was lowest (9%) at −0.6 V. The current peaks were higher at −0.7 V and at −0.8 V but also had a 14−20% coefficient of variation. The variation was even larger (39%) at −1.5 V although the peak current was higher. This steep change in current is due to the reduction of H_2_O + *e^−^* ⇋ H_2_ (*g*) + OH^−^ [48]. In addition, the signal variation became higher because ASV in the presence of trapped hydrogen bubbles degraded the Cu WE, as confirmed from visual corrosion of the electrode. We therefore settled for −0.6 V as the optimized potential with the lowest variation coefficient.

Another explored ASV parameter is the deposition time. Longer deposition (up to 15 min) usually yields stronger peak current and is recommended for sensing low concentrations of analyte [48]. However, thick RA foil electrodes could provide strong peak current even with short deposition. As results in Figure 4c show, peak current increases with deposition time from 10−50 s with <8% variation. In the tradeoff between faster process and lower LOD, we settled for 10 s deposition which resulted in a 260 nA peak current for 1 nM Pb in 0.2 M buffer. It should be noted that a thick-foil electrode such as the one fabricated in this work can support deposition time longer than 10 min, if desired, which is not often possible with thin-film electrodes due to oxidation and delamination issues [30].

The SWASV waveform parameters were also optimized. The peak current increases at higher square wave frequency and amplitude (Figure 4d−e). Higher pulse frequency produces a higher signal variation, although the variation was insignificant for pulse amplitude below 100 mV. For the pulse increment, a 10 mV step potential was used (Figure 4f). We therefore settled for 10 Hz, 100 mV, and 10 mV as the waveform frequency, pulse amplitude, and increment, respectively. These optimized values were then used in Pb ASV for calibration of the sensors. The experimental parameters are summarized in Table 2.

### 3.4. Voltammetric Measurements and Sensor Calibration

**Figure 5:**
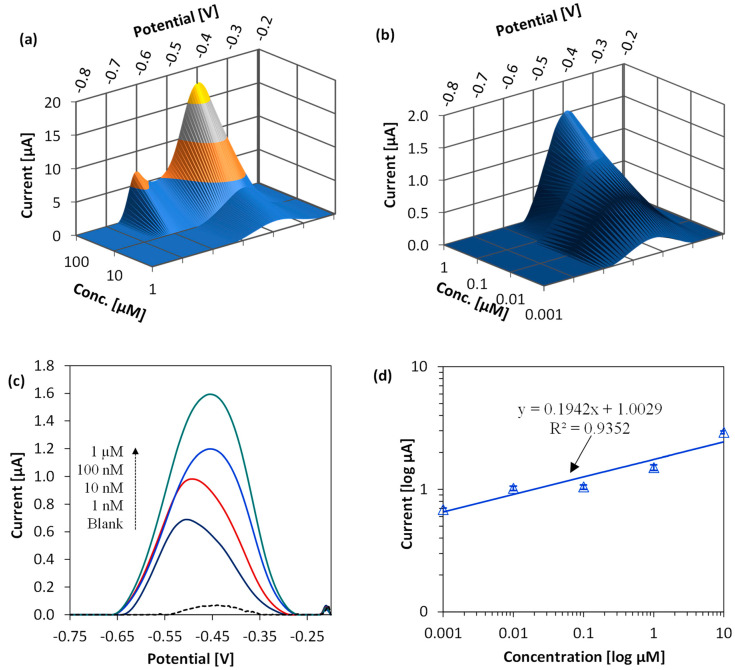
(**a**,**b**) ASV peak profile for 1 nM–100 µM Pb in deoxygenated acetate buffer (0.2 M, pH 5.2). (**c**) Anodic stripping voltammogram in 1 nM–1 µM Pb using parameters from Table 2. (**d**) Sensor calibration curve in buffer. Standard deviation obtained for n = 3.

Figure 5a,b show the stripping peak profile (split for better viewing) of Pb in deoxygenated acetate buffer. The ASV of 1 nM–10 µM Pb produced single stripping peaks, whereas Pb concentrations greater than 10 µM produced double peaks. As discussed, ASV of high Pb concentration registers a second current peak due to initial stripping of Pb atoms from the surface of the underlying Pb layer followed by stripping from the electrode surface. The ASV peak amplitudes in both low concentration and high concentration ranges increased linearly, as observed in the peak profile split views. This behavior is due to the mass transport characteristics of the system. The mass transport can be related to convection, migration, and diffusion [48]. Since the solution is not mechanically stirred within the PDMS well, convection does not occur and does not affect the formation of a diffusion layer on the working electrode. In addition, the effect of migration is eliminated by introducing a high concentration of excess acetate buffer that acts as an inert electrolyte. Therefore, diffusion is the significant form of mass transport for this integrated three-electrode system.

Figure 5c shows the ASV stripping peaks of varying Pb concentrations. The slight shift of voltammograms towards a more positive potential at higher Pb concentrations is due to the logarithmic dependence of electrochemical (electrode) potential on the analyte concentration [47] (Equation (1)), and it does not affect the peak amplitude [30]. Therefore, any potential shift should not be a major concern for disposable sensors if they are properly calibrated. The calibration curves in Figure 5d show that the fabricated sensors exhibit a slope of 0.1942 µA/µM. The calculated sensitivity is ~11 nA/ppb/cm^2^, given that the diameter of the electrode is 1.5 mm. If a higher peak current (higher sensitivity) is desired, a longer deposition time can be used (Figure 4c). For 1 nM–10 µM Pb in buffer, the correlation equation between peak current *I*(*μA*) and Pb concentration [*Pb*(*μM*)] is given by *I*(*μA*) = 0.1942 × [*Pb*(*μM*)] + 1.003 with R^2^ = 0.935. ASV of the smallest detectable Pb concentration (1 nM) produces a 688 nA peak current. By visual evaluation [49], the sensor LOD was estimated to be 0.2 µg/L (or 0.2 ppb).

Table 3 compares this work with other Pb sensors reported in the literature. In terms of analysis time required for Pb determination, this work features the fastest sensing (30 s) and a sub-parts-per-billion level LOD. The LOD of this work is an order lower than that of our previous work that used physically modified MWCNTs with β-cyclodextrin [21]. In the tradeoff between these two performance parameters, the use of rolled-annealed Cu foil electrodes offers the best combination.

**Figure 6:**
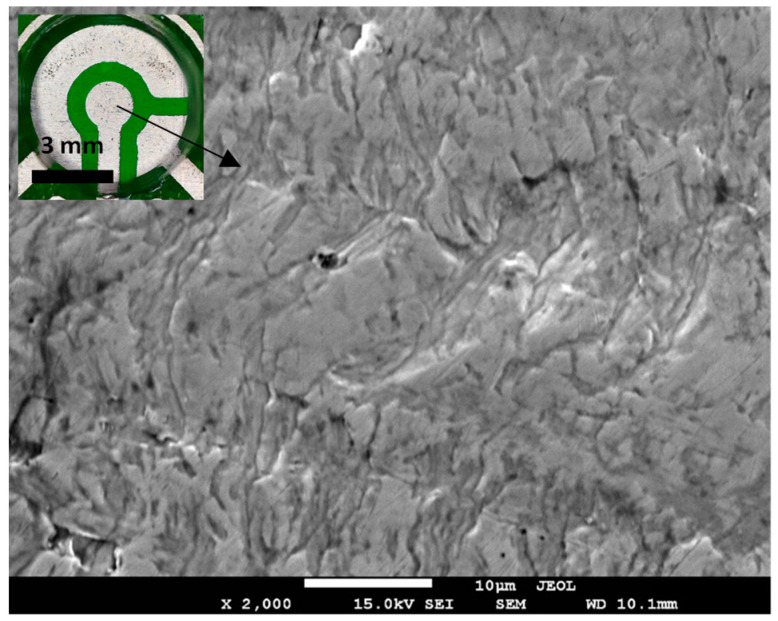
SEM micrograph of rolled-annealed Cu electrode surface. Inset shows a fabricated sensor.

Conventionally, a higher deposition time is desirable when the sensor cannot provide sufficient peak current due to low analyte concentration, small electrode surface area, or poor conductivity of the electrode [48]. To address these issues, deposition time, electrode material, and electrode geometry are carefully chosen. For example, using nanostructures such as single-wall carbon nanotubes (SWCNTs) [50] increases the effective surface area of the electrode, but at the cost of greater fabrication complexity. Comparing electrodes with the same geometry, rolled-annealed Cu electrodes may have better performance than electrodeposited Cu electrodes. Merchant et al. [34] characterized the cross sections of rolled-annealed and electrodeposited Cu and identified differences in their grain boundaries. When annealed, rolled Cu foil underwent recovery and recrystallization, forming compact columns of vertically aligned grains, whereas the grains in electrodeposited Cu were randomly oriented with weak crystallographic textures [34]. This recrystallization is linked to increased electrical conductivity of RA Cu at higher annealing temperatures [60], which may enable detection of small stripping currents from trace analyte. Figure 6 shows a scanning electron micrograph of the RA Cu-based WE used in this work.

### 3.5. Interference Study

**Figure 7:**
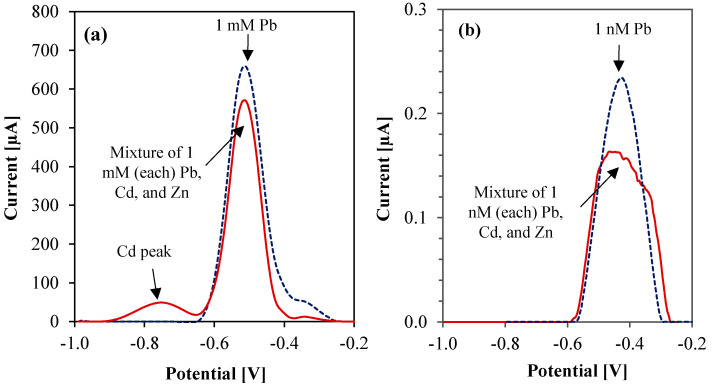
Interference study (ASV using parameters from Table 2) in acetate buffer (0.2 M, pH 5.2) with equally mixed concentrations of: (**a**) 1 mM Pb, Cd, and Zn (compared with 1 mM Pb alone), and (**b**) 1 nM Pb, Cd, and Zn (compared with 1 nM Pb alone).

We investigated the interference effects of Cd and Zn on the Cu-based sensor because these ions often coexist with Pb in water and their ASV peaks are separated by a few hundred millivolts [61]. Thus, these peaks may interfere with that of Pb. To assess the effect of interference from co-existing metals, we compared the sensor performance when both Cd and Pb are present in solution and its performance when only Pb is present. As shown in Figure 7a, ASV of equally mixed high concentrations (1 mM) of Pb, Cd, and Zn in 0.2 M, pH 5.2 acetate buffer registered two distinct peaks in contrast to a single peak for 1 mM Pb alone. Although one would expect three distinct peaks for Pb, Cd, and Zn, the relative oxidation potential of Zn is far more negative than that of Cd [30] and is therefore outside the observed potential window. Thus, Zn^2+^ is the least interfering ion in this study. This observation may seem obvious since the optimized deposition potential (−0.6 V) is not sufficiently negative [48] to reduce Zn^2+^ on the working electrode in the first place. The peak reduction of Pb for the mixture of 1 mM Pb, Cd, and Zn in Figure 7a however indicates that Cd causes interference. The 1 mM Cd oxidation peak at −780 mV is much smaller compared to the 1 mM Pb oxidation peak at −500 mV, which either signifies that the Cu working electrode has poor affinity towards Cd^2+^ ions, or the deposition potential used for Pb sensing causes only a partial deposition of Cd atoms on the electrode. Kang et al. [30] observed similar fractional deposition on the working electrode and explained it in terms of under-potential deposition. Therefore, careful optimization of the deposition potential may also reduce interference.

Next, we repeated ASV of equally mixed low concentrations (1 nM) of Pb, Cd, and Zn and compared it with that of 1 nM Pb alone. As results in Figure 7b show, we recorded peak distortion and broadening in the presence of 1 nM interfering ions, although the distinct Cd-oxidation peak was not observed.

**Figure 8:**
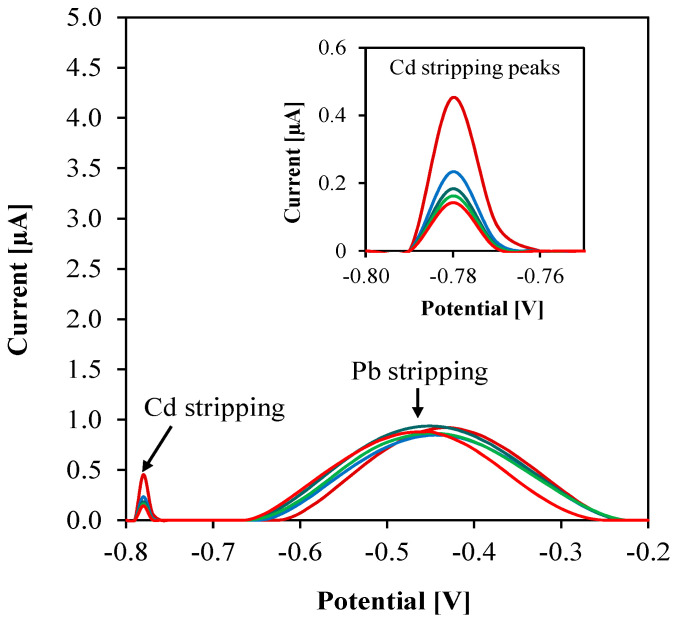
ASV of fixed 5 nM Pb with increasing concentrations of Cd in steps of 1 nM, 10 nM, 100 nM, 1 µM, 10 µM, and 100 µM using parameters from Table 3. Inset shows the Cd stripping peaks in magnified scale.

We further investigated the effect of varying Cd concentrations (1 nM−100 µM) while maintaining a fixed 5 nM Pb concentration in the samples. As results in Figure 8 show, all Cd stripping peaks were registered separately, and a tenfold increase in Cd concentration did not significantly affect the Pb stripping peaks. This signifies that the fabricated sensor can sensitively detect at least 1.0 µg/L Pb even in the presence of interfering ions. To conclude, the presence of Cd in drinking water may result in a reduction of peak current and a slight loss of peak resolution. However, since Pb and Cd commonly exist in surface water in the range of 5−30 µg/L and <1 µg/L respectively [30], we expect negligible interference on the sensor based on this ratio.

## 4. Conclusions

In this work, we developed a copper-based electrochemical sensor for trace lead detection. For the first time, we demonstrated the potential of rolled-annealed (RA) Cu foil to fabricate a three-electrode configuration bonded to a flexible polymer substrate. The mechanically flexible Cu/polymer bonded materials allowed for a single-step LaserJet print−and−transfer of a polyester resin-based mask on Cu thereby reducing sensor fabrication complexity, time, and cost. Additionally, the compact bulk grain structure and smoothness of RA Cu foil electrodes enabled the detection of electrolytic cell current in the range of a few nanoamperes with a very low coefficient of variation. Furthermore, the integrity of difficult-to-bond smooth sensing electrodes was achieved through a direct bonding process. Bonding between Cu and LCP was performed using a single-step, low temperature, low pressure, oxygen plasma-activated method that resulted in good adhesion (500 N/m). Investigation of Cu and LCP surfaces suggests that the bond formation is due to interdiffusion of thermally grown Cu_x_O nanoparticles across highly hydrophilic bonding surfaces.

In terms of sensing performance, the Cu-based sensor exhibited good linearity at very low Pb concentration levels. In addition to detecting Pb in only 30 s, the sensor provided a limit of detection of 0.2 µg/L (ppb). This was the first time that an integrated Cu-based sensor featured such as a rapid detection of such a low concentration of Pb. The sensor also showed robustness to interference from Cd and Zn. The presence of 1 nM Cd and Zn interfering ions slightly affected the amplitude and resolution of the 1 nM Pb ASV peak, but the distortion was mostly due to Cd. In surface water treatment applications where Cd concentration is much lower compared to Pb, we expect little to no interference. Additionally, we optimized the deposition potential for Pb^2+^ so that it is less electronegative than the potential required to deposit interfering ions. The test solutions used in this work were deoxygenated by nitrogen bubbling and a PDMS-based electrolytic chamber was introduced in the sensor to avoid distortion due to the presence of oxygen. However, the adhesion between the substrate and electrode is strong, making it useful for online tap water and remote water monitoring, particularly in situations where the water has dynamic flow. Further research is needed to examine the long-term stability and reliability of the sensor in real-world environments. We envisage interfacing this miniaturized sensor with an analog front-end integrated potentiostat such as the LMP91000 (Texas Instruments, Dallas, TX, USA) to realize a robust handheld Pb determination system.

## Figures and Tables

**Table 1:** Surface activated bonding (SAB) parameters for directly bonded electrodes.

Process Parameter	Value
RIE power (W)	100
RIE time (s)	240
O_2_ flow (sccm)	98
Vacuum Pressure (Pa)	100
Bonding temperature (°C)	230 for 1 h
Bond-head pressure (MPa)	0.3

**Table 2:** Square wave ASV parameters.

Process Parameter	Value
Deposition potential (V)	−0.6
Deposition time (s)	10
Pulse frequency (Hz)	98
Amplitude (mV)	100
Increment (mV)	10

**Table 3:** Comparison of this work with similar electrochemical Pb sensors.

Electrode Material			Electrode Fabrication	LOD (µg/L)	Deposition Time (s)	Reference
WE	CE	RE				
Rolled-annealed Cu foil	Cu	Cu/CuCl_2_	LaserJet printing of electrode mask on rolled-annealed Cu/polymer. Integrated metal foil-based microelectrodes bonded to a polymer substrate. RE fabricated by anodic chlorination of Cu using embedded electrodes.	0.2	10	This work.
MWCNTs with β-CD	Pt	Ag/AgCl	MWCNTs with β-CD drop casted on screen-printed carbon electrode.	0.9	600	Our previous work [21]
Bi-TRGO/Au	Au	Ag/AgCl	Photolithographic processing of fully-integrated microelectrodes on thermally-reduced graphene oxide on Si substrate followed by electrodeposition of Bismuth.	0.4	150	[1]
Cu thin-film	Cu	Cu/CuCl_2_	Fully-integrated thin-film electrodes formed by e-beam evaporation of Cu on adhesive Ti layer, followed by photolithography and Ti/Cu etch.	4.4	300	[30]
Bi-C	Pt	Ag/AgCl	Screen printing & electrodeposition of bismuth- coated carbon electrode.	0.3	120	[17]
Bi-nanopowder/Nafion	Pt	SCE	Dispersion of gas-condensed bismuth nanopowder on carbon with Nafion.	0.17–1.97	180	[18]
Bi	Pt	SCE	Screen printing and electrochemical reduction of Bi_2_O_3_.	2.3	300	[19]
Hg-Bi/SWCNT on GCE	Pt	Ag/AgCl	Single-walled carbon nanotube functionalization of glassy-carbon electrode by liquid drop followed by ex-situ chemical deposition of Hg and Bi metals.	1.2 × 10^−3^	300	[20]
Sb-boron doped diamond	C	SCE	Electrochemical modification of antimony nanoparticles on boron-doped diamond electrode.	18.5	120	[22]
SWCNT	Pt	SCE	Vacuum filtering of single-walled carbon nanotube on an anodic membrane followed by photolithographic processing.	0.8	150	[50]
Au	C	Ag	Screen printing of Au and Ag.	0.5	120	[51]
Bi-Nafion-graphene on GCE	Pt	Ag/AgCl	Glassy-carbon electrode with composite paste made by dispersion of graphene in Nafion solution and in-situ plating of Bi film.	0.5	120	[52]
Bi/Nafion/poly-pyrrole on GCE	Pt	SCE	Pyrrole polymerization followed by thiolene overoxidation & Nafion coat on glassy-carbon electrode.	0.05	300	[53]
Bi/GCE	Pt	Ag/AgCl	In-situ deposition of bismuth on glassy-carbon electrode.	0.8	120	[54]
Bi nano-hexagons on Cu	Pt	Ag/AgCl	Hexagon-shaped bismuth nano- and micro-architectures electrodeposition onto polycrystalline Cu film.	0.05	600	[55]
Boron doped diamond	Pt	Ag/AgCl	Synthesis of diamond films in a hot filament chemical vapor deposition reactor.	1	180	[56]
Bi/graphene-ionic composite	Pt	Ag/AgCl	Electrochemical reduction of graphene oxide on an ionic liquid followed by in-situ bismuth deposition to form a composite paste for electrode.	0.1	120	[57]
Bi-CNT	Pt	SCE	In-situ bismuth plating on screen-printed carbon nanotube electrode.	1.3	300	[58]
Porous Bi	Pt	Ag/AgCl	Electrochemical deposition of bismuth into a polystyrene-based nano-spherical porous template.	1.3	90	[59]

## Data Availability

The experimental data are available on request to corresponding author.
